# The complete chloroplast genome sequence of *Lycoris radiata*

**DOI:** 10.1080/23802359.2019.1660265

**Published:** 2019-09-10

**Authors:** Fengjiao Zhang, Xiaochun Shu, Tao Wang, Weibing Zhuang, Zhong Wang

**Affiliations:** Jiangsu Key Laboratory for the Research and Utilization of Plant Resources, Institute of Botany Jiangsu Province and Chinese Academy of Sciences (Nanjing Botanical Garden Mem. Sun Yat-Sen), Nanjing, China

**Keywords:** *Lycoris radiata*, chloroplast genome, phylogenetic analysis

## Abstract

*Lycoris radiata*, widely known as red spider lily. It belongs to the Amaryllidaceae family and has highly ornamental and medical value. Here, we assembled the complete chloroplast genome sequence by high-throughput sequencing and bioinformatics, which will provide more genomic information to the analysis of genetic diversity and phylogenetic relationship. The full length of chloroplast genome is 158,335 bp, composed of a large single-copy region (LSC) of 86,612 bp, a small single-copy region (SSC) of 18,261 bp, and a pair of inverted repeats (IR) of 26,731 bp. A total of 137 genes were annotated, including 87 protein-coding genes, 42 tRNA, and 8 rRNA genes. Phylogenetic tree analysis revealed that the close relationship between *L. radiata* and *L. squamigera* in the Amaryllidaceae family.

*Lycoris radiata* belongs to the genus *Lycoris*, comprised of approximately 20 species and limited distribution in eastern Asia (Meerow and Snijman 1998). The bulbs are rich in family-specific alkaloids for medicinal use (Martin 1987) and the flower is beautiful for ornamental plant. Due to the frequent interspecific hybridization and diverse intraspecific variation, many researchers have focused on the phylogenetic analysis in *Lycoris* (Lledó et al. 2004; Shi et al. 2006). *Lycoris radiata* was considered to be a probable ancestral species in the genus, but there is not sufficient evidence to support the point due to the abundant genetic variations, including triploid, diploid, and tetraploid population (Zhou et al. 2007). Some DNA sequencing method was performed to clarify the phylogenetic analysis in the genus (Roh et al. 2002; Hori et al. 2006), but complete chloroplast (cp) genome is rarely reported except for the *L. squamigera* (Jin et al. 2018). Here, we assembled the complete cp genome sequence of *L. radiata* by Illumina sequencing and bioinformatics analysis, which will provide more sequences data to understand the genome and phylogenetic relationship of the Amaryllidaceae family.

*Lycoris radiata* bulbs were planted in Nanjing Botanical Garden, Mem. Sun Yat-sen (E118_83, N32_06), Jiangsu province, China. The fresh leaves were collected for whole genome DNA extraction. The plant DNA isolation reagent (Code: D9194, TaKaRa, China) was used to extract DNA according to the instructions. Quality DNA was performed by Illumina sequencing with paired-end 150 strategy at Novogene company (http://www.novogene.com/). A total of 57.6 Gb reads were generated and 0.68 Gb reads were assembled with cp genome by the organelle assembler NOVOPlasty Version 3.3 (Dierckxsens et al. 2017). The *L. squamigera* cp genome (GenBank no. MH118290.1) was chosen as a reference (Jin et al. 2018). In the end, the cp genome annotation and visualization were performed using web server CPGAVAS2 (http://www.herbalgenomics.org/cpgavas2) (Shi et al. 2019). The *L. radiata* cp genome was deposited in GenBank (accession no. MN158120).

The complete cp genome size of *L. radiata* was 158,335 bp, which was composed of a large single-copy region (LSC) of 86,612 bp, a small single-copy region (SSC) of 18,261 bp, and two inverted repeat regions of 26,731 bp. A total of 157 genes were predicted in cp genome, including 107 protein-coding genes, 42 tRNA genes, and 8 rRNA genes. Among them, 18 splitting genes contained introns and exons and two of them (*ycf3*, *clpP*) had two introns, others had a single intron.

Phylogenetic analysis was conducted with cp genome of 16 species (including *L. radiata*) in five related families. The sequences were aligned by MAFFT online service (version 7) with default parameters (https://mafft.cbrc.jp/alignment/server/) (Rozewicki et al. 2019) and the phylogenetic tree was constructed using neighbor-joining (NJ) analysis with 1000 bootstrap replicates followed the manual of MAFFT online service (Figure 1). In the phylogenetic tree, all species were clustered on correct clades of family, *L. radiata* was grouped together with *L. Squamigera* and other seven species in the Amaryllidaceae family.

**Figure 1.**
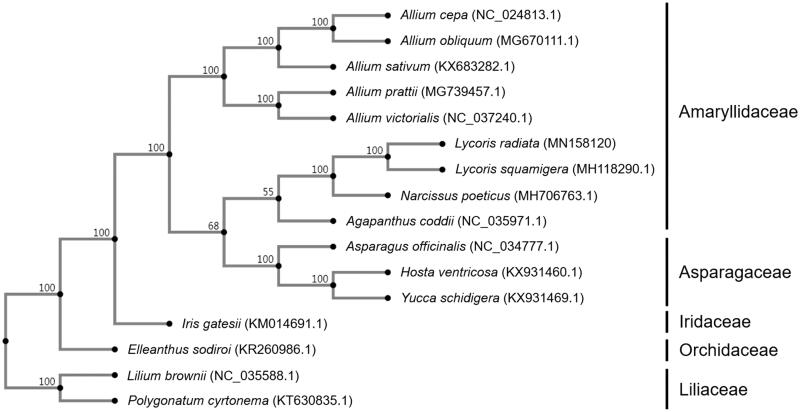
Phylogenetic tree based on the cp genome sequences of 16 species, showing the close relationship between *L. radiata* and *L. squamigera*. Numbers next to the nodes indicate the bootstrap value from 1000 replicates. Genbank accession no. of each species was shown in the brackets after names.
